# Strength of Evidence on Demand Creation for Voluntary Medical Male Circumcision From 7 Impact Evaluations in Southern and Eastern Africa

**DOI:** 10.1097/QAI.0000000000001161

**Published:** 2016-10-06

**Authors:** Eric W. Djimeu, Annette N. Brown

**Affiliations:** * International Initiative for Impact Evaluation (3ie), Washington, DC; and; † Department of Research and Evaluation Strategic Initiative, FHI 360, Washington, DC.

**Keywords:** HIV, Africa, male circumcision, demand creation, impact evaluation, evidence, financial compensation, influencers

## Abstract

Seven new impact evaluations of pilot programs for increasing the demand for voluntary medical male circumcision (VMMC) provide evidence of what works and what does not. The study findings suggest that financial compensation designed to relieve the opportunity or transportation costs from undergoing the procedure can increase the uptake of VMMC. There is also evidence that programs using peer influence can be effective, although so far only sports-based programs demonstrate a strong effect. We explore the strength of evidence in each of these 7 studies to better interpret the findings for policy making.

**Methods:**

We perform a risk of bias assessment and conduct power calculations using actual values for each of the 7 studies.

**Results:**

Three of the 7 studies have a medium risk of bias, whereas the other 4 have a low risk of bias. All but 2 of the studies have adequate power to detect meaningful effects. In the 2 with insufficient power, the estimated effects are large but statistically insignificant.

**Conclusion:**

The positive evidence that financial incentives presented as compensation for opportunity costs to men seeking and obtaining VMMC can increase uptake comes from strong studies, which have high power and low to medium risk of bias. The positive evidence that a comprehensive sports-based program for young men can increase uptake also comes from a strong study. The strength of the studies further validates these findings.

## INTRODUCTION

Since 2007, despite several initiatives to increase the prevalence of male circumcision in the 14 countries prioritized by the WHO and UNAIDS, the progress has been modest relative to the goal. Although the goal was to circumcise 20 million men by 2015 to have a high epidemiologic impact,^1^ as of the end of 2015, just over 10 million circumcisions were completed in the these countries.^2^

Behavior change communication (BCC) approaches to increase demand have been largely used.^3^ BCC is “the strategic use of communication to promote positive health outcomes”^4^ using a systematic process that involves individuals and communities. BCC can use a variety of channels, including mass media and community mobilization, to provide information on benefits from voluntary medical male circumcision (VMMC). BCC approaches alone are not enough to increase demand; a trial evaluating the impact of comprehensive information about male circumcision and HIV risk in Lilongwe, Malawi revealed that the information increased the likelihood of getting circumcised by only 1.4% points after 1 year.^5^

Acceptability studies suggest that cost might be a barrier, both the costs of undergoing the procedure, which may include transportation cost to the clinic and the price of the procedure, and the opportunity costs including time away from work during the procedure and healing.^6^ A randomized controlled trial (RCT) conducted in Kenya of an intervention to reduce opportunity costs associated with VMMC finds that small, fixed economic incentives to compensate for lost wages ranging from KES 700–1200 (USD 8.75–15) increased VMMC uptake within 2 months among men aged 25–49 years by 7.1%.^7^

In this article, we review 7 recent impact evaluations that tested a variety of innovative strategies for increasing the demand for VMMC.^8–14^ Several of the interventions include aspects of BCC, with 2 relying primarily on BCC (SMS information in Zambia, information postcards in South Africa), and others combining information with influencers (intimate female partners in Uganda, male peers in Zambia, male role models in Zimbabwe, and emotional framing messages that play on intimate partner preferences and masculinity in South Africa). Two studies explore direct financial incentives (food vouchers for circumcision in Kenya and transportation allowance for VMMC counseling in South Africa). These BCC and financial compensation interventions address identified barriers to VMMC. Two additional innovative pilot interventions tested whether men might respond to material incentives through lotteries that provided smartphone prizes to the winners (one offered a selection of a smartphone or bicycle).^11,12^

Although these studies were conducted at roughly the same time and were designed with counterfactuals, the interpretation of the results should take into account the strength of evidence considering specific features of the evaluations. In this article, we explore the strength of evidence for each of the 7 impact evaluations by performing a risk of bias assessment and conducting power calculations with the actual values of the relevant parameters. We then interpret the findings across the 7 studies taking into account the relative strength of evidence.

## EVALUATION METHODS

International Initiative for Impact Evaluation (3ie) defines impact evaluation as that which measures an attributable net impact of a program or intervention using a counterfactual. These 7 impact evaluations serve implementation science objectives as they examine how to best promote VMMC. The use of counterfactual methods as part of the implementation science produces strong evidence of what does and does not work and provides estimates of net effects that allow for cost-effectiveness analysis, which is crucial to making resource allocation decisions. Six of the impact evaluations were designed originally as RCTs; 3 of these as cluster RCTs with 1 randomizing across schools and 2 randomizing across health facilities. The seventh was designed as a quasi-experimental study using a natural experiment (timing of the third trimester of pregnancy) to produce the counterfactual.

## REVIEW METHODS

We use a risk of bias assessment tool developed to assess impact evaluation studies included in systematic reviews.^15^ The tool critically appraises the likely risk of bias based on: (1) whether the attribution methods address confounding and sample selection bias; (2) whether possible spillovers to comparison groups are addressed; (3) the presence of outcome and analysis reporting bias; and (4) the presence of other sources of bias.^15^ The tool instructs the user to assign scores of yes, unclear, or no for each of the 4 characteristics. This particular tool has detailed descriptions for assessing the quality of attribution methods for both experimental and quasi-experimental designs. The tool describes what should be considered low, medium, and high risk of bias.^15^ We use those descriptions to assess overall risk based on the scores for the 4 characteristics.

In each of the evaluations, we calculate the actual power for estimation to inform the interpretation of findings. The protocol for each of these studies did include standard power calculations using assumed values of parameters to determine sample size. The power calculations we present here use the true values of these parameters determined through the actual conduct of the interventions. Such “*ex-post*” power calculations account for sample attrition, actual coefficient of variation, actual intracluster correlation (if applicable), and actual share of the sample in the treatment group. This exercise is particularly useful when comparing findings across multiple studies.^16^ The formulas used to perform these power calculations come from Djimeu and Houndolo.^17^ Power is calculated using one-sided statistical tests because we assume that the alternative hypothesis is expected to be unidirectional.

## RESULTS

Table 1 presents the main features of the 7 impact evaluations in this supplement, including the final attribution method and the actual sample size. See Sgaier et al^18^ for short descriptions of each intervention. The qualitative studies in this supplement are not reviewed here.

**TABLE 1.**
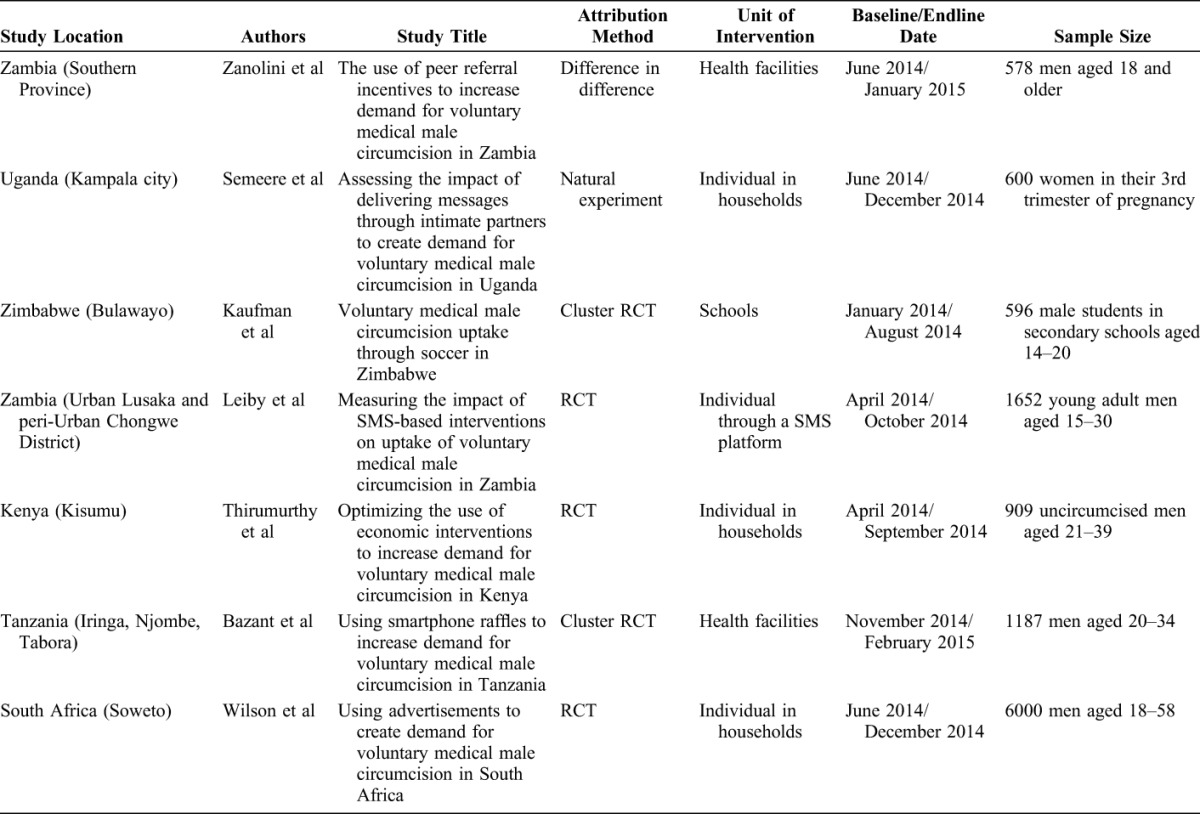
Features of 7 Impact Evaluations of VMMC Demand Creation

After implementation, 5 evaluations maintain randomization as their attribution method, and 2 use a quasi-experimental method. The peer referral incentive in Zambia, designed as a cluster RCT, was converted to a quasi-experimental design during implementation because of problems with low power and need to add additional study facilities. The study uses facility data from before and after the intervention to calculate the difference-in-difference effect for treatment facilities compared with nontreatment facilities.

According to its design, the study of messaging through intimate partners in Uganda uses a quasi-experimental method. The natural experiment takes advantage of the randomness of when women happen to be in their third trimester of pregnancy. The researchers observed circumcisions among partners of a sample of women in their third trimesters attending antenatal care clinics for 3 months to serve as the control group. After a 2-month wash out period to prevent contamination, the team implemented the intervention in women in their third trimesters attending antenatal clinics during a 3-month period.

### Risk of Bias Assessment

All 7 evaluations use a counterfactual design to address selection bias and measure an attributable impact. The findings can still be subject to many other sources of bias, both anticipated and unanticipated in the evaluation design. Table 2 presents the results of the risk of bias assessment.

**TABLE 2.**
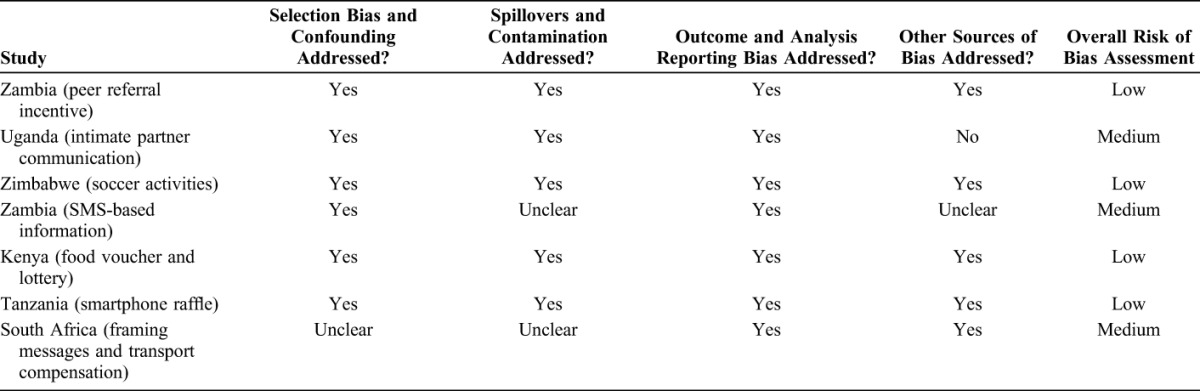
Risk of Bias Assessment for 7 Impact Evaluations of VMMC Demand Creation

Of the 7 studies, 3 have medium risk of bias. For the Uganda study, we see a potential source of bias because of the control phase and intervention phase occurring during different times of year. Evidence from other studies suggests that seasonality affects the demand for male circumcision in many of the priority countries.^19^ In the case of Uganda, the control period was May to August 2014, and the intervention period was October 2014 through January 2015. The latter is a period of many end-of-year festivities and the research team in Uganda explained in our communications with them that circumcision rates tend to drop during these festivities. Seasonality could thus bias the measured effect down, and this potential source of bias is not addressed in the study.

We score 2 characteristics as “unclear” for the study of SMS messaging in Zambia. The authors acknowledge that the SMS surveys may suffer from survey contamination. One man could give his phone to a friend and let the friend answer the questions. Similarly, there are possible spillovers from leaked messages—when one man shows the messages on his phone to one or more of his friends. The inability of the researchers to verify who is being surveyed and who is receiving the intervention when both are performed through text messaging makes it unclear whether contamination and spillovers are fully addressed. A bigger issue may be social desirability bias in the self-reported uptake data. The authors find a high inconsistency between the self-reported uptake data and uptake verified through registers. Although the inconsistency is not correlated with the treatment arms, it is still a cause for concern. We thus score unclear for whether other sources of biases are fully addressed.

For the South Africa study, the study team randomized the allocation of postcards by randomizing the order of the postcards within each set of 6 possible cards and then having outreach workers distribute 1 postcard to each fifth house. Five houses may not be far enough apart to prevent spillovers. Someone with a control card or a framing message card who was more likely to get circumcised could obtain a financial compensation card from a neighbor and bias the results in favor of the effectiveness of the financial compensation relative to the messaging and control. There is also possible selection bias if someone who does not get a postcard but who was already planning to get circumcised obtains a financial compensation postcard from a neighbor. Because the circumcision is not a result of the incentive, this would bias up the uptake of the financial compensation relative to a random distribution of cards.

### Power Calculations

Table 3 presents the power calculations using the actual values of the parameters from each evaluation. For this table, we present separate rows for the studies that produce an effect size for more than 1 intervention type, specifically for the compensation and lottery interventions in Kenya and the compensation and framing message interventions in South Africa. The measured effect for the Zambia peer referral evaluation is the percentage increase in monthly circumcisions, whereas the other effects are percentage point differences in uptake, or in the case of Tanzania, the percentage point difference in the uptake increases. The minimum detectable effect estimated in the fifth column is the smallest effect size the study could have measured given the realized parameters and holding power at 80%. The power presented in column 6 is how much power the study has, given the realized parameters, for the actually measured effect to be statistically significant.

**TABLE 3.**
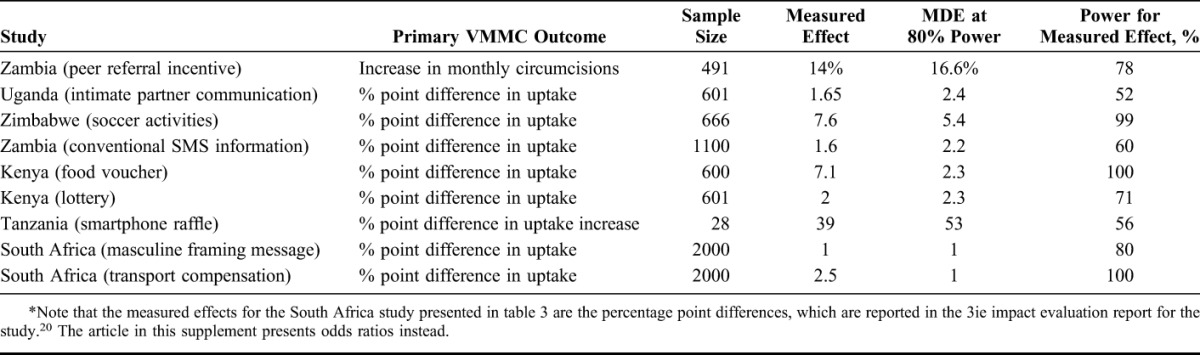
Power Calculations for Minimum Detectable Effect (MDE) and Statistical Power for 7 Impact Evaluations of VMMC Demand Creation*

The Uganda intimate partner study and the Zambia SMS messaging evaluations, both of which assigned treatment at the individual level, had 80% power to detect fairly small effect sizes, 2.4% and 2.2% points, respectively. This means that the sample sizes were big enough to measure statistically significant differences between uptake in the intervention group and control group of 2.4% and 2.2% points. The measured effects were even smaller, however. The Zambia peer referral study and the Tanzania study were both cluster designs, which reduces power *ceteris paribus*. Based on their sample sizes, the minimum detectable effects at 80% power were relatively high, 16.6% increase in monthly circumcisions for facilities in Zambia and a 53% point difference in the increase in circumcisions for facilities in Tanzania. The measured effect in Zambia came close; the study found a 14% increase in monthly circumcisions. In Tanzania, the effect measured by the study was 39% points, a large effect but below the minimum detectable difference. Note, however, that the effects across the 7 pairs of facilities varied greatly. The percentage increase in the number of circumcisions in the intervention facilities ranged from −29% to 218%; in the control facilities from −71% to 500%.

The Zimbabwe evaluation used an RCT design and had 80% power to detect a percentage point difference in uptake of 5.4. The Kenya evaluation used an RCT design and could detect differences as low as 2.3% points. Both evaluations measured impacts over 7% points (just for the food voucher arm in Kenya). The South Africa study, which randomized at the individual level and had a very large sample relative to the other studies, was powered to detect effects as small as 1 percentage point and did measure small effects with statistical significance.

## DISCUSSION

Considering both risk of bias and power, the 2 studies with the strongest positive results are the Zimbabwe soccer-based intervention and the Kenya financial compensation (food voucher) intervention. Both studies have a low risk of bias and high power. In both cases, the intervention produced a rate of circumcisions over 7% points greater than in the control groups.

The results from the 2 financial compensation studies, Kenya food voucher and South Africa transport voucher, provide strong evidence that fixed financial compensation can increase VMMC uptake. The Kenya food voucher study has high power and a low risk of bias. The South Africa transport voucher study has high power but a medium risk of bias where the measured effect could be increased if the men seeking the transport voucher cards would have been circumcised regardless. We do not consider the risk of bias to be a serious problem for the South Africa study, particularly as the finding that financial compensation is effective is consistent with the Kenya food voucher finding. Note that the South Africa intervention paid the transport reimbursement just for receiving the counseling, so the compensation may not need to be tied directly to circumcision.

The 2 tests of lottery-based material incentives are less encouraging. The Kenya study is powered at 80% to detect an effect as low as 2.3% points but did not find a statistically significant effect. Although the Tanzania study measured a large effect, it was statistically insignificant as the study was not well powered. Both studies have a low risk of bias, so bias does not call the findings into question.

The intervention tested in Zambia combines elements of 2 other interventions with strong positive evidence, a small financial incentive and peer influencers. The study has a low risk of bias, and although the measured effect is large, there is insufficient power to detect a statistical significance suggesting that further consideration of this or a similar intervention is warranted.

The Uganda study of the intimate partner intervention and the Zambia study of SMS messaging both have sufficient power to measure relatively small differences in uptake and yet neither found a statistically significant effect. Both also have a medium risk of bias. The possible source of bias for the Uganda study, that the timing of the intervention during months when circumcision uptake is low, would bias the effect down and could account for the null result. However, given the qualitative evidence showing that most women did not feel comfortable engaging their partners in a conversation about circumcision, the null result is likely valid. The risk of bias in the Zambia SMS study comes from measurement problems, as well as from possible spillovers. The study team was careful to address the problems as much as possible in their analysis such that bias should not lead to questioning the null result.

In sum, the positive findings on financial compensation and the sports-based intervention come from strong studies, further validating those results. The finding on peer referral incentives in Zambia is promising and worth testing again with a higher-powered study. After consideration of the possible weaknesses in the other studies, we believe that the null results stand.
